# Natural killer cell engagers: From bi‐specific to tri‐specific and tetra‐specific engagers for enhanced cancer immunotherapy

**DOI:** 10.1002/ctm2.70046

**Published:** 2024-10-29

**Authors:** An Zhu, Yu Bai, Yanyang Nan, Dianwen Ju

**Affiliations:** ^1^ Department of Biological Medicines & Shanghai Engineering Research Center of Immunotherapeutics Fudan University School of Pharmacy Shanghai China; ^2^ Shanghai Institute of Infectious Disease and Biosecurity Fudan University Shanghai China

**Keywords:** bi‐specific NKCEs, cytokines, natural killer cell engagers, NK receptors, tetra‐specific NKCEs, tri‐specific NKCEs

## Abstract

Natural killer cell engagers (NKCEs) are a specialised subset of antibodies capable of simultaneously targeting endogenous NK cells and tumour cells, generating precise and effective cytolytic responses against cancer. This review systematically explores NK engagers as a rising star in NK‐mediated immunotherapy, specifically focusing on multi‐specific engagers. It examines the diverse configuration of NKCEs and how certain biologics could be employed to boost NK activity, including activating receptor engagement and cytokine incorporation. Some challenges and future perspectives of current NKCEs therapy are also discussed, including optimising pharmacokinetics, addressing the immunosuppressive tumour microenvironment and exploring potential combinatorial approaches. By offering an in‐depth analysis of the current landscape and future trajectories of multi‐specific NKCEs in cancer treatment, this review serves as a valuable resource for understanding this promising field of immunotherapy.

**Highlights:**

- **Innovative NKCEs**: NK cell engagers (NKCEs) represent a promising new class of immunotherapeutics targeting tumours by activating NK cells.
- **Multi‐specific formats**: The transition from bi‐specific to multi‐specific NKCEs enhances their versatility and therapeutic efficacy.
- **Mechanisms of action**: NKCEs have the potential to improve NK cell activation by engaging activating receptors and incorporating cytokines.
- **Clinical potential**: Current clinical trials demonstrate the safety and efficacy of various NKCEs across different cancer types.
- **Future research directions**: Optimising NKCE designs and exploring combination therapies are essential for overcoming challenges in cancer treatment.

## Graphical abstract

- Multi‐specific NKCEs: Multi‐specific NK cell engagers (NKCEs) enhance the immune system's ability to target and destroy tumor cells.
- Diverse targeting: These NKCEs can engage multiple antigens on NK cells and tumor cells, providing a more robust cytotoxic response.
- Enhanced cancer immunotherapy: The multi‐specific design of these NKCEs offers improved effectiveness in cancer immunotherapy, potentially leading to better clinical outcomes.

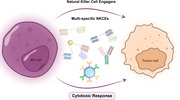

## 1 INTRODUCTION

Natural killer (NK) cells are a critical component of the innate immune system. They can rapidly eliminate malignant cells without prior sensitisation and have been recognised as a potential candidate for cancer immunotherapy. In recent years, the development of natural killer cell engagers (NKCEs) has emerged as one of the groundbreaking advancements in harnessing this innate power to combat tumours. NKCEs are specialised antibodies engineered to simultaneously target endogenous NK cells and tumour cells, forming a bridging effect that enhances the natural cytotoxicity of NK cells.^1^, ^2^ The type of NKCEs has progressively developed over the several decades, from initial bi‐specific designs to more complex multi‐specific formats. Currently, over 10 different NKCEs are in clinical trials, and over 30 types of NKCEs are in developing stages,^3^, ^4^, ^5^, ^6^ underscoring the growing interest and potential of this therapeutic strategy.

By simultaneously engaging multiple targets, multi‐specific NKCEs are able to provide higher versatility and efficiency than traditionally bi‐specific NKCEs.^7^ This multi‐pronged approach potentially leads to more robust NK cell activation and higher binding specificity and affinity to cancer cells,^2^, ^5^, ^8^ partially addressing the challenges faced by earlier antibody therapies, such as high off‐targets rate and low responsive rate.^9^, ^10^

This review explores NKCEs in cancer immunotherapy, with a particular focus on multi‐specific NKCEs. We discuss NK cell biology and the principles underlying NKCEs design, including the various formats such as bi‐specific, tri‐specific and tetra‐specific engagers. The review examines the clinical applications of NKCEs, detailing ongoing trials and emerging results across different cancer types. We also investigate strategies to enhance NK cell activity through NKCEs. Finally, we offer perspectives on the future of NKCEs in cancer treatment, discussing challenges, opportunities and potential combinatorial approaches to optimise their therapeutic efficacy.

## 2 BASIC OF NK CELLS

**FIGURE 1:**
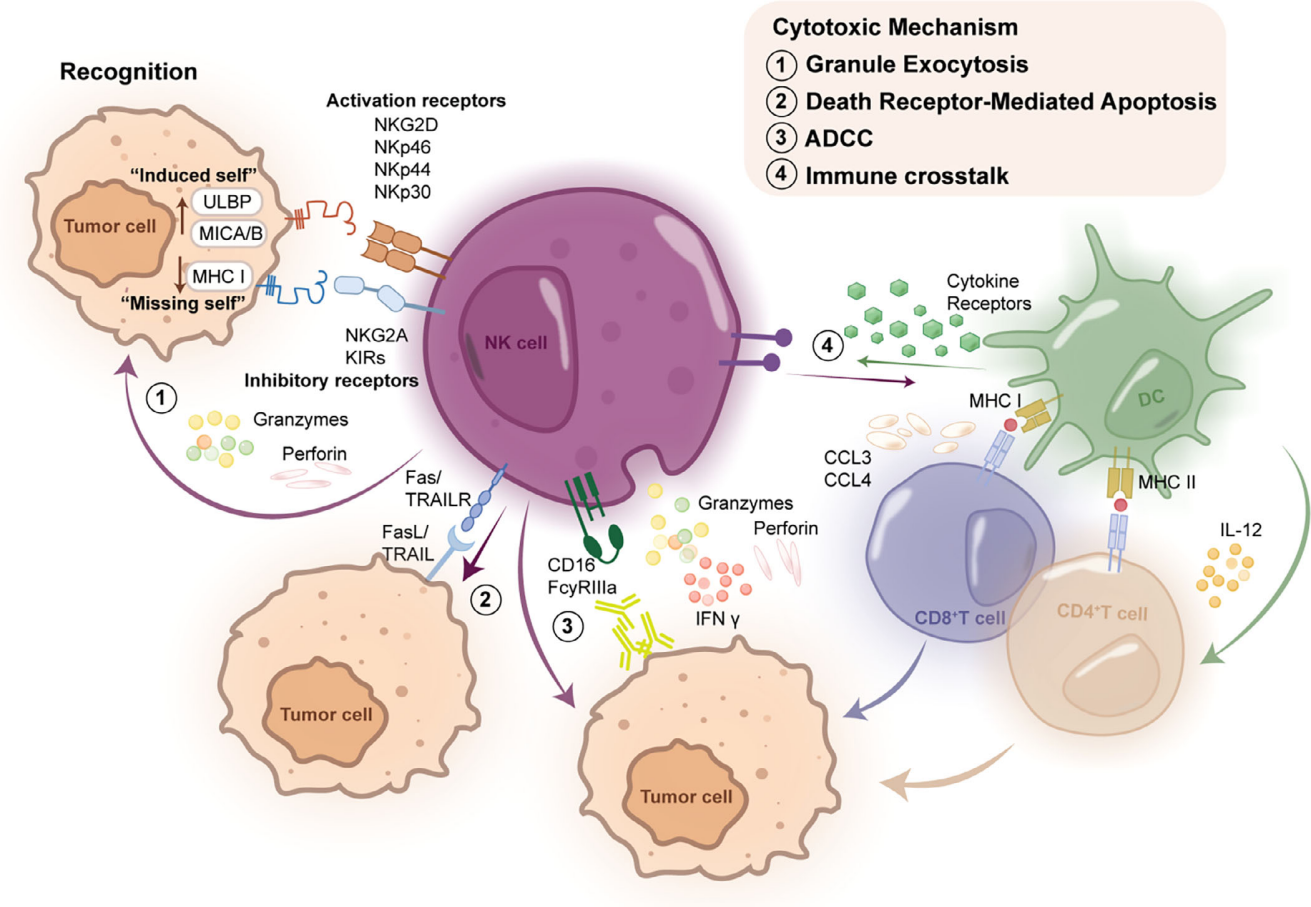
NK cell functions in tumour recognition and elimination. This figure illustrates the integrated processes by which natural killer (NK) cells recognise and eliminate tumour cells. NK cell activation: Cells utilise a balance of activating and inhibitory receptors to detect tumour cells. Activating receptors (e.g., NKG2D, NKp46, NKp44, NKp30) recognise stress‐induced ligands (e.g., ULBP, MICA/B) on tumour cells (‘induced self’). Inhibitory receptors (e.g., KIRs, NKG2A) interact with MHC I molecules, often down‐regulated in tumour cells (‘missing self’). The integration of these signals determines NK cell activation. Cytotoxic mechanisms: Upon activation, NK cells employ multiple, simultaneous strategies to eliminate tumour cells. 1: Granule exocytosis: Release of perforin and granzymes, leading to target cell lysis and apoptosis. 2: Death receptor‐mediated apoptosis: Expression of FasL and TRAIL, which bind to death receptors on tumour cells, inducing apoptosis. 3: Antibody‐dependent cell‐mediated cytotoxicity (ADCC): CD16 (FcγRIIIa) on NK cells binds to antibody‐coated tumour cells, triggering cytotoxic responses. Cytokine production: Activated NK cells produce cytokines, primarily IFN‐γ, enhancing anti‐tumour immune responses and modulating the tumour microenvironment. 4: Immune crosstalk: NK cells interact with other immune cells, such as dendritic cells (DCs) and T cells, through cytokine production and cell‐cell contact, amplifying the overall immune response against tumours.

NK cells are a heterogeneous population of lymphocytes derived from the common lymphoid progenitor cells in the bone marrow. Unlike T cells, NK cells can rapidly respond to stressed or abnormal cells without prior sensitisation. The innate ability and lower risk of triggering cytokine storms make NK cells particularly attractive for cancer immunotherapy strategies.^9^ Based on their expression of CD56, NK cells could be divided into two distinct subpopulations, CD56^dim^ and CD56^bright^, reflecting distinct maturation stages and receptor profiles. CD56^dim^ NK cells are predominantly found in peripheral blood and exhibit potent cytotoxicity, while CD56^bright^ NK cells are predominantly enriched in lymphoid tissues with enhanced migratory capacity and primarily engage in cytokine production.^10^ This functional dichotomy underscores the diverse roles of NK cells in immune surveillance and regulation across various anatomical compartments. NK cells express various receptors, including natural cytotoxicity receptors (NCRs) and killer cell immunoglobulin‐like receptors (KIRs). These receptors are important in recognising ligands expressed on the surface of target cells (Table 1). The delicate equilibrium between inhibitory and activating signals originating from these receptors dictates the activation state of NK cells. Under normal conditions, healthy cells express high levels of Major Histocompatinility Complex class I (MHC I) molecules on their surface, which serve as ligands of inhibitory receptors, tipping the balance towards NK cell inactivation. However, when the healthy cells transform into abnormal cells, their surface ligands expression can vary. For example, to evade T cell‐mediated killing, breast cancer cells will down‐regulate Human Leukocyte Antigen‐A (HLA‐A) expression.^11^ Conversely, many cancer cells, including colorectal and lung cancer cells, up‐regulate the expression of activating ligands such as MHC class I polypeptide‐related sequence A/B (MICA/B).^12^ These changes alter the balance of signals received by NK cells, potentially leading to their activation (Figure 1). Once recognition is completed, the NK cell will adhere closely to the tumour cell, establishing a cytolytic immune synapse (IS), then releasing cytotoxic granules containing perforin and granzymes to lyse the tumour cells. The precise cell‐killing mechanism is ensured by the highly organised structure of the cytolytic IS.^13^ In addition, NK cells also secrete cytokines and chemokines that can influence the immune response by recruiting dendritic cells (DCs) and T cells to the tumour microenvironment (TME).^13^ By secreting chemokines such as CCL5, XCL1/2 and FLT3LG, NK cells facilitate the recruitment of conventional type 1 DCs (cDC1s), a specialised subset of DCs that participate in the cross‐presentation of tumour antigens to CD8+ T cells.^14^ This process coordinates a multicellular immune response against the tumour, potentially enhancing the efficacy of immunotherapy. Another cytotoxic mechanism of NK cells is antibody‐dependent cellular cytotoxicity (ADCC) (Figure 1). This process is mediated primarily by the Fc‐gamma receptor immunoreceptor tyrosine‐based activation motif (ITAM) (CD16a), with FcγRIIb and FcγRIIc, which are found in some NK cell subsets.^15^ Notably, CD16a is also expressed on monocytes and macrophages, enabling antibody‐dependent cellular phagocytosis which expands the range of antibody‐mediated immune responses.^15^ In this process, CD16a receptors on the NK cells will identify complexes formed by tumour antigen‐bound IgG1 and IgG3 antibodies, then secrete perforin and granzymes to initiate apoptosis in tumour cells. Additionally, pro‐inflammatory cytokines, such as IFN‐γ and TNF‐α will be secreted when ADCC is activated, further enhancing the immune response against the target cells.^16^ Overall, the unique properties of NK cells make them attractive candidates for cancer immunotherapy, and numerous strategies have been developed to harness their anti‐tumour activity.

**TABLE 1:** Inhibitory and activating receptors on the surface of natural killer cells targeted by NKCEs.^17^, ^18^, ^19^, ^20^, ^21^

Family	Activating receptor	Ligands
Immunoglobulin superfamily receptors KIR (killer immunoglobulin like receptors)	KIR2DS1 KIR2DS2 KIR2DS3 KIR2DS4 KIR2SD5 KIR3DS1	HLA‐C2 HLA‐C1 Unknown HLA‐C (subset) /HLA‐F Unknown HLA‐F
Immunoglobulin superfamily receptors SLAM	2B4 (CD244)	CD48
Immunoglobulin superfamily receptors NCR	NKp44 NKp30 NKp46	HLA‐DP, PDGF‐DD, 21spe‐MLL5, Nidogen 1 B7‐H6, BAT3 Viral hemagglutinins, heparan sulphate, proteoglycans, GFP and vimentin
Immunoglobulin superfamily receptors Others	DNAM‐1 CD16a (Fc gamma receptor III)	PVR/Nectin‐2 IgG1 and IgG3 isotype
C‐type lectin‐like receptors	NKG2D NKG2C/CD94 NKp80	MICA, MICB, ULBP1‐4, ULBP1‐6 HLA‐E ACLL, CLEC2
Family	Inhibitory receptor	Ligands
Immunoglobulin superfamily receptors KIR (killer immunoglobulin like receptors)	KIR2DL1 KIR2DL2 KIR2DL3 KIR2DL4 KIR2DL5 KIR3DL1 KIR3DL2(subset of NK)	HLA‐C2 HLA‐C1 HLA‐C1 HLA‐G PVR HLA‐Bw4 HLA‐A3, HLA‐A11, HLA‐F
Immunoglobulin superfamily receptors ILT/LIR	LIR‐1	HLA‐A, ‐B and ‐C
C‐type lectin‐like receptors	NKG2A/CD94	HLA‐E
Others	CD160	HLA‐C

PVR, poliovirus receptor.

## 3 ENHANCING NKCEs FUNCTIONALITY

In cancer immunotherapy, the effectiveness of NKCEs is crucial and depends on various factors, including the formation of optimal ISs and their ability to target tumours. Improving the functionality of NKCEs requires strategic advancements. Key aspects include the selection of appropriate NK cell receptors, the incorporation of immune checkpoints and cytokines and remodelling of the microenvironment. These strategies, which will be explored in the following sections, are part of a broader effort to optimise NKCEs performance and expand their therapeutic potential.

### 3.1 NK cell receptors

The activation of NK cells hinges on the equilibrium between signals from activation receptors and inhibitory signals. While both inhibitory and activation receptors can theoretically serve as candidates, current trends favour activation receptors. Choosing an appropriate receptor is critical for building an NKCE, the influence factor including the availability and distribution of these activation receptors as well as their physical structure, since some receptors have protruding shapes while others are not.

#### 3.1.1 CD16a

CD16a, known as Fcγ receptor IIIa (FcγRIIIa), is an essential activating receptor primarily found on CD56^dim^ NK cells.^16^ This receptor binds to IgG antibodies, initiating a signalling cascade involving enzymes like PI3K, phospholipase Cγ and Vav, which lead to NK cell activation. Notably, CD16a can trigger the complete activation of NK cells independently, distinguishing it from some T cell receptors.^22^ A notable molecule that incorporated CD16a is AFM24, a bi‐specific NKCE that targets the EGFR on solid tumours. Preclinical findings suggested that this molecule obtained high‐affinity binding to CD16a and showed therapeutic effectiveness in various EGFR‐expressing tumour cells.^23^

To develop CD16a NKCEs, an important aspect that needs to be considered is the rapid shedding of CD16a from NK cells post‐activation, triggered by metalloproteases (e.g., ADAM17) and matrix metalloproteases (e.g., MMP25).^24^ Introducing MMP inhibitors alongside CD16a‐specific NKCEs is an avenue being explored to curb CD16a removal from NK cells, potentially heightening their responsiveness.^25^ It is also essential to realise that NKCEs aimed at CD16a might attach to the CD16b receptors found on neutrophils due to their notable similarity to their extracellular domain to CD16a on NK cells.^26^ Moreover, the soluble CD16 in the bloodstream, largely derived from CD16b from neutrophil surfaces and a lesser amount from CD16a of NK cells, could lead to a significant ‘sink’ effect.^27^ This phenomenon might contend with CD16a on NK cells for NKCEs binding, potentially hampering their efficiency. Potential methods to enhance the specificity of CD16a include engineering antibodies with higher affinity, epitope mapping for unique CD16a binding sites or employing multi‐specific antibodies.^28^, ^29^

#### 3.1.2 NKG2D

NKG2D (KLRK1) belongs to the lectin‐type receptor family. It is notable for its distinct signalling pathway that does not intersect with the inhibitory signals of KIRs, thereby reducing the likelihood of its activation being hindered by inhibitory ligands.^30^, ^31^ Human NKG2D receptors differentiate themselves by not associating with ITAMs or ITAM‐bearing adaptors; instead, they exclusively bind with the adaptor protein DAP10.^31^, ^32^, ^33^ Instead of following the ITAM‐adaptor‐induced signalling, DAP10 fosters the recruitment and activation of PI3K and a molecular complex of Grb2 and Vav1.^34^ NKG2D can bind to several MHC‐I‐like ligands (Table 1). While these ligands generally have a low presence on healthy cells, their expression can be amplified due to infections or oncogenic transformations. This ligand‐binding capability of NKG2D on NK cells leads to its cytotoxic prowess. Yet, its capacity to spur cytokine secretion remains an area of ambiguity.^35^ Although monoclonal antibody‐induced crosslinking of NKG2D does not trigger cytokine release, stimulation with its soluble ligands has demonstrated the release of cytokines like Interferon‐γ(IFN‐γ), Granulocyte‐Macrophage Colony‐Stimulation Factor(GM‐CSF) and Macrophage Inflammatory Protein‐1 beta (MIP‐1β.^36^ Moreover, the functional capacity of NKG2D can be further fine‐tuned by IL‐15, which prompts phosphorylation of DAP10 via Jak3, a prerequisite for recruiting the p85 subunit of PI3K and Grb2.^37^ However, the consistent engagement of NKG2D with its ligands can lead to a down‐regulation, which may result in NK cell desensitisation.^38^ Another layer of complexity arises from the existence of soluble forms of ligands. These can be shed from the tumour cell surface or secreted via exosomes, but their biological roles are yet to be fully deciphered.^39^

#### 3.1.3 NKp30

NKp30, or NCR3 or CD337, is a type I transmembrane activating receptor belonging to the immunoglobulin superfamily. This receptor is constitutively expressed on both naïve and activated NK cells, ILC2, subsets of T cells (CD8+ T cells, γδ T cells).^40^ Mediating its signalling through association with ITAM‐bearing molecules (CD3ζ and FcRγ), NKp30 involves in the induction of NK cell cytotoxicity and cytokine secretion.^41^ NKp30 interacts with a diverse array of structurally dissimilar ligands, including the tumour‐specific surface molecule B7‐H6, the nuclear factor BAT3 released from tumour cells, and various viral and parasitic proteins.^42^ The presence of soluble B7‐H6 in the blood of cancer patients has been associated with NKp30 down‐regulation in tumour contexts, underscoring the significance of ligand–receptor interaction in disease prognosis.^43^ Immature DCs can also boost NK cell activity through NKp30 signalling, further enhancing their cytotoxicity.^44^ Another ligand for NKp30 is galectin‐3, a β‐galactoside binding protein that is highly expressed in many cancer cells.

#### 3.1.4 NKp46

NKp46, a prominent NCR, is consistently expressed on mature NK cells and ILC1. Recent studies suggest its expression extends to certain subsets of ILC3, particularly in mucosal tissues and on specific T lymphocyte populations. Structurally, NKp46 exhibits similarities with NKp30, with the distinction of possessing two extracellular Ig domains.^42^ While the precise ligands for NKp46 remain elusive, numerous potential ligands, inclusive of certain bacterial entities, have been proposed in literature.^45^ Signal transduction via NKp46 is predominantly mediated through FcRγ and the ITAM‐bearing molecule CD3ζ.^46^ A depth of research underscores the role of NKp46 in mediating NK cell‐induced lysis of diverse cell types, encompassing autologous, allogeneic and xenogeneic cells.^46^ Notably, the activation potential of NKp46 on resting NK cells remains suboptimal unless there is concurrent engagement with additional factors, such as 2B4, DNAM1 or CD2. Unlike CD16a, its expression will not be rapidly down‐regulated when NK cells are activated in several cancers such as lung carcinoma and AML.^47^, ^48^ With advancements in immunotherapy, the antibody‐based NK cell engager technology (ANKET) primarily focuses on targeting the NKp46 receptor.^2^

#### 3.1.5 NKG2C

NKG2C, also designated as CD159c, is an activation receptor with specificity for HLA‐E. This receptor is mainly expressed on the surface of mature NK cells that lack NKG2A.^49^ Elevated expression of NKG2C is indicative of an NK cell adopting a ‘memory’ phenotype.^50^ NKG2C can elicit a robust NK cell immune response independently without necessitating cooperation with other molecules. Distinct from CD16a, NKG2C does not undergo rapid shedding following NK activation.^51^, ^52^ Presently, NKG2C serves as a focal target in the design of tri‐specific killer engagers (TriKEs) aimed at combating acute myeloid leukaemia.^53^ However, a potential limitation of this receptor is its comparatively low and variable expression level across NK cells relative to CD16a.^54^ Past research has highlighted that individuals with prior exposure to human cytomegalovirus typically exhibit elevated proportions of NKG2C^+^ NK cells.^55^

In the domain of NKCEs development, a critical consideration is the trade‐off between the advantageous and disadvantageous properties of each receptor. Contemporary research has begun to converge on the strategy of dual receptor incorporation within a single NKCE framework, thereby augmenting binding specificity and attenuating potential off‐target interactions. A notable example of receptor integration is the synergistic pairing of CD16, which can induce strong ADCC but is susceptible to shedding after activation, with NKp46, which remains stable post‐activation but lacks the capacity to trigger cytokine secretion independently.

### 3.2 Incorporating cytokine and immune checking points

The presence of stimulatory cytokines, specifically IL‐2 and IL‐15, has been observed to augment NK cell proliferation and cytotoxicity while also enhancing the expansion of CD8+ T memory cells.^56^ Notably, approximately 50% of Tri‐specific NKCEs encompass stimulatory cytokines. Although IL‐2 immunotherapy boosts T and NK cell response, its clinical application is restricted by its toxicity.^57^ Integrating IL‐2 into NKCEs helps balance toxicity and immune cell enhancement. Nevertheless, it is imperative to consider the down‐regulation of CD16 expression observed with prolonged IL‐2 exposure.^58^ This phenomenon could potentially compromise the efficacy of numerous NKCEs that primarily target CD16 as the only activation receptor. Compared with IL‐2, IL‐15 exhibits analogous functions but with milder effects, and to enhance their potency, it is commonly built into complexes with (IL‐15/IL‐15Rα) to mimic the cellular trans‐presentation of IL‐15.^59^

In certain malignancies, tumours can modulate the expression of inhibitory receptors or their ligands to evade anti‐tumour responses. For instance, non‐small cell lung cancer (NSCLC) cells often up‐regulate PD‐L1, which binds to PD‐1 on T and NK cells, inducing their exhaustion and suppressing anti‐tumour activity. This mechanism enables tumours to escape immune surveillance and proliferate.^60^ Such alterations can compromise the affinity between NKCEs and their intended targets. Checkpoint inhibitors aimed at PD‐1 and CTLA4 have notably advanced the ability to counteract T‐cell tolerance in various cancers.^61^ Concurrent studies suggest that this might extend to NK cells, given their expression of PD‐1.^62^

### 3.3 Bi‐specific NKCEs

Bi‐specific killer cell engagers (BiKEs), exemplified by AFM13, AFM28 and AFM24, are at the forefront of cancer immunotherapy innovation. Several other BiKEs are also progressing alongside these leaders, marking a significant advance in cancer treatment strategies. These treatments, each uniquely targeting specific cancer antigens, have shown distinct mechanisms of action but also face certain limitations. AFM13, a chimeric Tandem Diabody (TandAb), combines a mouse‐derived anti‐CD30 binding domain with a human anti‐CD16a domain to activate potent ADCC. Its effectiveness, observed in a Phase I trial for CD30‐positive Hodgkin lymphoma, was modest, likely due to impaired autologous NK cell functions in patients. This limitation is being addressed by exploring the combination of AFM13 with allogeneic NK cells, showing promising early results in treating CD30‐positive blood cancers.^7^, ^63^AFM28 targets CD123 and is part of the Redirected Optimized Cell Killing (ROCK) platform (Table 2). Designed as an Fc‐silent monoclonal antibody with anti‐CD16 (scFv) at the ends of the heavy chain, it effectively triggers ADCC in AML cell lines and blocks IL‐3R signalling, currently being tested in a Phase I trial with AML patients.^64^, ^65^ Despite their innovative design and potential for effective cancer treatment, BiKEs confront challenges. These include limited target antigen specificity, leading to non‐specific toxicity and antigen escape mechanisms like NKG2C hiding. Their small size often results in a short circulation time in the body, and the effectiveness of NK cells can be affected by the immunosuppressive TME.

**TABLE 2:** Overview of major NK cell engagers production platforms.^2^, ^6^, ^7^, ^66^, ^67^

Platform	Developer	Technical features	Structure	Mechanism of action	Representative products	Advantage
BiKE	Affirmed	TandAb technology	Tetravalent bi‐specific	Simultaneous binding to CD16a and tumour antigen	AFM13 (CD30/CD16a), AFM24 (EGFR/CD16a)	High affinity, longer half‐life
TriKE	University of Minnesota & GT Biopharma	Includes IL‐15 domain	Tri‐specific	Binds NK cells, tumour cells and stimulates NK cell proliferation	GTB‐3550 (CD16/IL‐15/CD33)	Mediates contact and stimulates NK cell proliferation
ANKET	Innate Pharma	Dual NK cell receptor targeting	Tri/tetra‐specific	Enhances NK cell activation via CD16 and NKp46	IPH6101 (NKp46/CD16 /CD123)	Multiple activation receptor binding, increased specificity
TriNKET	Dragonfly Therapeutics	Triple targeting	Tri‐specific	Targets tumour antigen, CD16 and NKG2D	DF1001 (HER2/CD16 /NKG2D)	Multiple mechanisms for NK cell activation
ROCK	Affirmed	Fc‐ optimisation	Fc‐based antibody	Enhances NK cell binding and activation through optimised Fc	AFM24 (EGFR/CD16a)	High design flexibility

## 4 MULTI‐SPECIFIC NKCEs

### 4.1 Tri‐specific engagers with dual tumour antigen targeting

**FIGURE 2:**
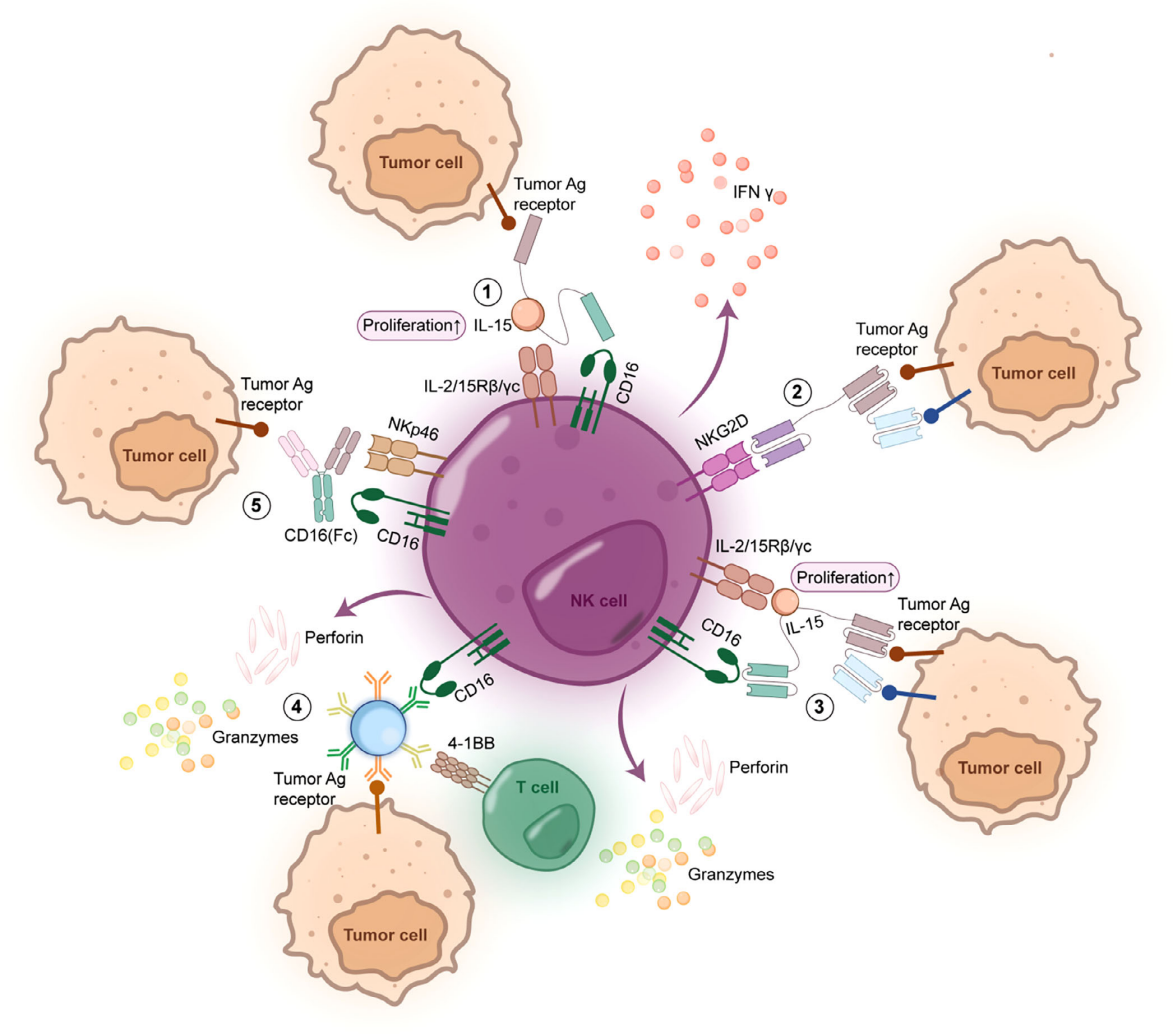
Various forms of tri‐specific NKCEs amplify the anti‐tumour effect of NK cells. This diagram represents five distinct types of tri‐specific NK engagers designed to augment the interactions between NK cells and tumour cells. (1) An IL‐15‐based TriKE, the IL‐15 addition promotes the proliferation and activation of NK cells. (2) NKCEs that dual targeted the antigens on tumours enhanced the binding specificity. (3) Tetra‐specific NKCEs dual‐targeted the tumour cells with IL‐15 incorporated. (4) Specialised nanoparticle‐based engagers capable of targeting either NK cells or T cells. (5) Dual‐targeted NKCEs that stimulate two NK cell activating receptors. Each engager operates through different mechanisms to synergistically boost the immune system's ability to recognise and destroy tumour cells.

Bi‐specific engagers have demonstrated promising results in treating lymphatic system malignancies like AML.^68^, ^69^ However, as research delves deeper, certain limitations have become evident, including reduced plasma retention and a higher off‐target rate. To overcome these weaknesses, the bi‐specific engagers and the triple component bi‐specific engagers (two scFvs binding to the same antigens such as (CD33/CD16/CD33) were generated (Table 3).^70^ Increased anti‐tumour activity generated by ADCC was observed in these molecules. The inaugural tri‐specific NK engager CD123/CD16/CD33 was constructed in a triplebody format. This single‐chain polypeptide molecule uniquely combined three scFvs, wherein each fragment exhibited its specificity, setting it apart from prior three‐component bi‐specific engagers.^71^ Compared with earlier bi‐specific engagers, the tri‐specific ‘triplebody’ molecules amplify the anti‐tumour effect by dual targeting. Simultaneously binding to two antigens on the same cell decreases the likelihood of off‐target interactions, enabling it to address antigen loss variants like mixed lineage leukaemia.^72^ Compared with two bi‐specific engagers (CD123/CD16/CD123 and CD33/CD16/CD33), the highest averaged maximum lysis was observed in the patients with CD123/CD16/CD33 treatment.^71^ This molecule brings the NK engagers into the new era, and many other tri‐specific engagers were designed with similar ideas. In addition to focusing on the CD16 receptors, several dual antigen‐binding NKCEs are designed with the NKG2D. More recently, a trifunctional NKCE targeted NKG2D/ULBP2–aCD19–aCD33 was reported to combat chronic lymphocytic leukaemia. In this study, the dual‐targeting format, ULBP2–aCD19–aCD33, demonstrated promising anti‐tumour activity against primary CLL cells, in either allogeneic or autologous settings. Furthermore, this molecule exhibited efficacy even if one of the target antigens (CD19 or CD33) was not present on the tumour cell, providing a potential safety net against antigen loss variants commonly seen after targeted therapies.^72^ An innovative platform employing tri‐specific IgG‐like VHH‐based NKCE was generated to simultaneously target NKG2D on NK cells and HER2 and EGFR antigens on breast cancer cells.^73^ Figure 2 displays some forms of tri‐ and tetra‐specific NKCEs.

**TABLE 3:** Multi‐specific NKCEs in the development stage.^3^, ^74^, ^75^, ^76^

Category	Name	Structure	Specificity	Conditions
CD16	Triplebody	(scFv)3	CD16/CD33/CD19	Mixed lineage leukaemia
	Triplebody	(scFv)3	CD16/CD123/CD33	Acute myeloid leukaemia
	Triplebody (SPM‐2)	(scFv)3	CD16/CD123/CD33	Acute myeloid leukaemia
	TriKE	(scFv)3	CD16/CD22/CD19	ALL, B‐CLL and AML
	ATriFlex	scFv–diabody–scFv	CD16a/CD200/BCMA	Multiple myeloma
	HLE‐nano‐BiKE	(VHH)3	CD16/CD38/HSA	Multiple myeloma
	Nanoengager	Nanoengager	CD16/4–1BB–EGFR	EGFR^+^ cancer, lung cancer
CD16 Cytokine	*Trike (GTB‐3550)	scFv/IL‐15/scFv	CD16/IL‐15/CD33	Acute myeloid leukaemia/MDS
	TriKE	scFv/IL‐15/scFv	CD16/IL‐15/EpCAM	Various carcinomas
	TriKE	scFv/IL‐15/scFv	CD16/IL‐15/CD133	Cancer stem cells
	TriKE	scFv/IL‐15/scFv	CD16/IL‐15/CD19	B‐cell chronic lymphocytic leukaemia
	TriKE	VHH/IL‐15/scFv	CD16/IL‐15/B7H3	Ovarian cancer
	TriKE	VHH/IL‐15/scFv	CD16/IL‐15/HER2	Ovarian cancer
	TriKE	VHH/IL‐15/scFv	CD16/IL‐15/CLEC12A	Acute myeloid leukaemia
	TriKE	VHH/IL‐15/scFv	CD16/IL‐15/STEM8	Non‐small cell lung cancer
	TriKE	VHH/IL‐15/VHH	CD16/IL‐15/PD‐L1	Non‐small cell lung cancer
	TeraKE(TstAb)	scFv/IL‐15/scFv/scFv	CD16/IL‐15/EpCAM/CD133	Colorectal cancer
NKG2C	TriKE	scFV‐IL‐15/scFv	NKG2C/IL‐15/CD33	Acute myeloid leukaemia
NKp46	ANKET	Fab/Fc/Fab	NKp46–NKp30/ CD16/CD19–CD20	B‐cell acute lymphoblastic leukaemia
	ANKET4	Fab/Fc/Fab	NKp46/CD16/CD20/IL‐2v	B‐cell acute lymphoblastic leukaemia
	ANKET	Fab‐Fc‐Fab	NKp46/CD16/CD19–CD20–EGFR	Non‐Hodgkin's lymphoma
	ANKET	Fab/Fc/Fab	NKp46–NKp30/CD16/CD19–CD20	B‐cell acute lymphoblastic leukaemia
Check point/CD16	TsAb	Bs IgG‐Fab	EGFR/CD16a/PD‐L1	Epidermoid carcinoma
NKG2D	DuoBody (DB)–VHH (TtsAb)	bs IgG‐(VHH)2	HER2/cMET/EGFR–IL6R–NKG2D	Breast cancer
	TriNKET	Undisclosed	NKG2D/CD16/undisclosed antigen on tumour	Hemic and lymphatic disease
	SEEDbody	IgG‐like VHH‐based	EGFR/HER2/NKG2D	Breast cancer
	Triplebody	ULBP2–scFv–scFv	NKG2D/CD19/CD33	Mixed lineage leukaemia

### 4.2 Tri‐ and tetra‐specific NKCEs incorporated dual activation receptors

To fully activate NK cells and promote potent anti‐cancer immune responses, simultaneous engagement of multiple cell‐surface receptors is required. Exploiting this principle, various tri‐specific NK cell engagers (NKCEs) have been designed to target two receptors on NK cells simultaneously. NKp46 and CD16 are two frequently chosen combinations. NKp46, predominantly expressed on NK cells, maintains stable expression in various cancers. In contrast, CD16, also found on macrophages and monocytes, is rapidly shed upon NK cell activation. This combination leverages the specificity of NKp46 and the potent activation through CD16, potentially enhancing NK cell‐mediated anti‐tumour responses.^77^ Gauthier et al.^78^ conducted groundbreaking research on multi‐specific NKCEs. They developed tri‐functional NKCEs targeting CD19 or CD20 while activating NKp46 or NKp30 alongside CD16 to treat paediatric B‐cell acute lymphoblastic leukaemia (B‐ALL). In vivo, tri‐specific NKCE demonstrated superior efficacy compared with the either BiKEs or the combine therapy. In vitro, the CD19–NKp46–CD16 NKCE effectively activated resting NK cells from healthy donors and transplant recipients, enabling them to target and eliminate various leukaemia cell lines and primary blasts, including the NK cell‐resistant MHH‐CALL‐4.^2^

SAR4433597 (CD123/NKp46/CD16), the first ANKET to enter clinical trials, targets relapsed or refractory acute myeloid leukaemia (Table 4). Preclinical data demonstrated the efficacy of CD123‐NKCE in cancer intervention, leveraging its binding to NKp46 to enable NK cells to target malignancies effectively while avoiding complications associated with CD64, which impedes ADCC. Compared with the widely used Fc‐engineered ADCC‐enhancing IgG1 antibody, CD123‐NKCE showed superior potency across various experimental settings, unaffected by the presence or absence of CD64 on cancer cells. Furthermore, CD123‐NKCE's therapeutic potential is highlighted by its ability to eliminate CD123‐positive cells without triggering a concerning cytokine response or exhibiting harmful toxicity, positioning it as a promising candidate with both efficacy and safety attributes.^78^ Tri‐specific NKCE therapies (TriNKET) is another unique approach for crafting dual antigen‐binding multifunctional NKCEs. DF1001 is a notable agent from this platform and is the first TriNKET that entered the clinical stage, specifically aimed at HER2 while simultaneously engaging with CD16 and NKG2D.^6^ Currently, a Phase 1/2 trial is underway to assess the safety and potential effectiveness of this HER2‐focused TriNKET in treating patients with advanced HER2 positive solid tumours.

**TABLE 4:** Bi‐specific and tri‐specific NK engagers in clinical trials.

Name	Specificity	Cancer type	Phase	Trial number	Treatment approach
AFM13	CD30/CD16a (tetravalent)	TCL	II	NCT04101331	Monotherapy
		HL	I	NCT01221571	Monotherapy
		R/R HL	II	NCT02321592	Monotherapy
			I	NCT02665650	Combined with pembrolizumab
			I	NCT04074746	Combined with other NK cell therapy
AFM24	EGFR/CD16a (tetravalent)	Solid tumours	I/II	NCT04259450	Monotherapy
AFM26	BCMA/CD16a	R/R MM	I/II	NCT04434469	Monotherapy
AFM28	CD123/CD16a	R/R acute MM	I	NCT05817058	Monotherapy
RO7297089	BCMA/CD16a	R/R MM	I	NCT04434469	Monotherapy
DF1001	HER2/CD16/NKG2D	HER2+ solid tumour	I/II	NCT04143711	Combined with pembrolizumab
DF9001	EGFR/NKG2D/CD16	Advanced solid tumours	I/II	NCT04143711	Combined with nivolumab
DF2001	NKG2D/CD16/CD33	Acute myeloid leukaemia	I	NCT04789655	Monotherapy
DF3001	NKG2D/CD16/BCMA	Multiple myeloma	I	NCT04975399	Monotherapy
SAR443579	CD123/NKp46/CD16	AML, MDS	I/II	NCT05086315	Monotherapy
IPH64	NKp46/CD16/BCMA	R/R MM	I/II	NCT05839626	Monotherapy

Abbreviations: TCL, T‐cell lymphoma; HL, Hodgkin's Lymphoma; R/R MM, relapsed/refractory multiple myeloma; AML, acute myeloid leukaemia; MDS, myelodysplastic syndromes.

GTB‐3550 was terminated due to the development of GTB‐3650.

Recently, ANKET‐based tetra‐specific NKCEs were reported, engaging two NK receptors, NKp46 and CD16, β‐chain of IL‐2R and CD20 (Table 3). This innovative design achieves synthetic immunity by using an IL‐2 variant (IL‐2v) that does not bind to CD25, aiming to limit Treg activation while redirecting IL‐2 activity to NK cells via cis‐engagement of NKp46, CD16a and IL‐2Rβ, and by creating a specialised connection between NK and tumour cells, facilitating targeted destruction of the latter. Incorporating IL‐2v into the tetra‐specific ANKET primarily targets NK cells, enhancing efficacy at low doses and inducing unique NK cell responses. Furthermore, this ANKET encourages the development of the memory‐like CIML NK phenotype, known for its heightened functionality.^79^ However, notably, the dynamic expression of CD25 on NK cells may influence this approach's efficacy. Cytolytic NK cells up‐regulate CD25 upon activation, while CD56^bright^ NK cells constitutively express it.^10^ This suggests potential preferential targeting of CD56^dim^ NK cells and introduces complexity to ‘super’ IL‐2 engager therapies, warranting further investigation into NK cell subset responses and activation states.

### 4.3 IL‐15 incorporated tri‐specific NKCEs

#### 4.3.1 First‐generation TriKEs and first TetraKE

TriKE typically refer to a subgroup of tri‐specific NKCEs that incorporate IL‐15. IL‐15‐integrated NK engagers do not strictly qualify as tri‐specific, given that one of their components is a cytokine. The design of 161533 TriKE (CD33/IL‐15/CD16) was grounded on the previously established BiKE structure, targeting CD33/CD16. Compared with the BiKE molecules, 161533TriKE demonstrated enhanced NK cell cytotoxicity, degranulation and cytokine production against targets. Additionally, it improved NK cell longevity and growth. In an immunodeficient mouse model with HL‐60‐Luc tumours, this TriKE displayed superior anti‐tumour effects. It sustained human NK cell survival for at least 3 weeks in vivo, the group treated with BiKE (CD33/CD16) was merely detectable.^67^ The EpCAM (CD326), a glycoprotein around 40 kDa, is a potential therapeutic target of cancers as this molecule is commonly expressed in the carcinoma of the colon, ovary, breast and prostate. An earlier developed BiKE combined scFvs targeting both CD16 and EpCAM, exhibiting augmented in vitro NK cell cytotoxicity against prostate, breast, colon and head and neck cancers, even at minimal effector: target ratios.^80^ Subsequently, an EpCAM TriKE was introduced, incorporating an IL‐15 segment between the anti‐CD16 and anti‐EpCAM scFvs. This construct not only amplified cytolytic and cytokine responses but also bolstered NK cell proliferation relative to its EpCAM BiKE counterpart. Beyond EpCAM^+^ tumour cells, there's growing interest in targeting cancer stem cells (CSCs) that express CD133 due to their potential role in initiating cancer.^81^ Schmohl et al. engineered a BiKE with specificity for CD16 and CD133 present on carcinoma cells. To optimise its therapeutic potential, they further refined this BiKE into a CD16/IL‐15/CD133 TriKE 1615133 TriKE using DNA shuffling and ligation techniques. Compared with the previous generation, 1615133 TriKE demonstrated enhanced NK cell‐mediated cytotoxicity and significant NK cell expansion. Further amplification in anti‐cancer efficacy was observed upon incorporation of the IL‐15 crosslinker. When assessing NK cell‐related cytokine release, IFN‐γ levels associated with TriKE were higher than those with BiKE.^82^ Pioneering advancements in cancer immunotherapy witnessed the development of the first TetraKE. Rooted in the foundational principles of the bi‐specific NKCE platform, this innovative construct amalgamated four distinct functional entities: EpCAM, CD133, IL‐15 and CD16. Preliminary in vitro studies delineated the robust capability of TetraKE in cancer therapeutics. Notably, its simultaneous targeting of both EpCAM and CD133 epitopes provides a dually specific attack on tumour cells and the pivotal CSCs. This intricate design of TetraKE showcased superior therapeutic potential compared with its predecessors, the anti‐EpCAM BiKE and anti‐CD133 BiKE.^83^

#### 4.3.2 Second‐generation TriKEs

The first generation of TriKEs demonstrated promise; however, complications arose due to the incorporation of variant IL‐15 rather than the recombinant human wild‐type IL‐15. Prior studies have shown that recombinant human wild‐type IL‐15 is more effective in its standalone form than when the mutant IL‐15 is incorporated into the 161533 TriKE. Furthermore, some clinical trials involving systemic IL‐15 administration have reported expansion of CD8 and γδ T cells, resulting in unintended immunological consequences.^67^ This complication was rooted in the utilisation of scFvs. Such a configuration predisposed the structure to challenges such as aggregation and steric hindrance. To ensure the functional integrity of TriKEs, there was a need to modify the IL‐15 to be compatible with the structural nuances^67^ .To address these structural challenges, a single‐domain antibody sequence, often referred to as sdAb or VHH, was introduced as an alternative to the scFv anti‐CD16 binding domain. Distinctively, this sdAb, with its exclusive variable heavy domain, mitigates the risk of aggregation associated with the VL domain of an scFv, guaranteeing the appropriate conformation of the TriKE. This design of sdAb drew inspiration from specific segments of a Llama‐derived anti‐CD16 antibody. Subsequently, it was integrated into a humanised sdAb blueprint, denominated as ‘cam16’. Considering the modifications, the second‐generation camel‐derived (cam)16‐wtIL‐15‐33 TriKE outperforms its first‐generation counterpart. It manifests enhanced proliferative activity on NK cells, and in experimental settings, shows superior tumour suppression in preclinical murine models.^67^ During the evaluation of the 161533 TriKE (CD33/IL‐15/CD16), researchers identified off‐target effects on conventional myeloid and myeloid progenitor cells that were characterised by CD33 expression.^29^, ^84^ In response, a second‐generation TriKE was designed, targeting the CLEC12A antigen (CLEC12A/IL‐15/CD16), as a potential therapeutic for AML. Significantly, CLEC12A is distinctly expressed in AML cells and LSCs, yet it remains absent in regular haematopoietic stem cells (HSCs), minimising the potential for collateral toxicity.^85^, ^86^ Preliminary studies revealed that the CLEC12A TriKE proficiently directs NK‐cell‐mediated cytotoxicity towards CLEC12A‐expressing AML cells, in an antigen‐dependent fashion. Notably, the CLEC12A TriKE fosters a unique proliferation in NK cells and enhances their cytolytic activity against AML cells in vivo. Post CLEC12A TriKE treatment, HSCs exhibited a sustained ability to generate colonies, emphasising the minimal perturbation this immunotherapeutic approach inflicts on normal haematopoiesis. Moreover, after treatment with the CLEC12A TriKE, HSCs showed superior replication efficacy than their CD33 TriKE‐treated counterparts.^87^

The advanced second‐generation TriKE architecture has been applied to combat solid tumours. B7‐H3, an immune checkpoint protein, is frequently overexpressed in numerous solid tumours, including ovarian, prostate and lung cancers.^88^, ^89^ Capitalising on the refined TriKE methodology, a cam16/IL‐15/B7‐H3 TriKE was devised targeting ovarian and lung cancers. Preliminary in vitro studies revealed that the cam16/IL‐15/B7‐H3 TriKE offered superior cytokine signalling specificity to NK cells when juxtaposed with prior BiKE iterations, thereby eliciting a robust NK‐mediated cytotoxic response. This conferred a statistically significant augmentation in the NK‐mediated cytotoxicity against ovarian, prostate and lung cancer cells in vitro. Additionally, in xenograft ovarian mouse models, the administration of cam16/IL‐15/B7‐H3 TriKE displayed enhanced NK cell‐mediated suppression, particularly in ovarian cells with diminished CD16 expression.^90^

### 4.4 Other tri‐specific NKCEs

Targeting the PD‐L1/PD‐1 axis has shown success in impeding cancer cell immune evasion, and inhibiting PD‐L1 can restore NK cell function. A multi‐functional NKCE, PD‐L1/CD16a/IL‐15, was engineered to target PD‐L1‐positive cancer cells. This chimeric molecule effectively activated NK cells and recruited T cells to eliminate PD‐L1‐overexpressing tumours in vitro. In mouse models, the fusion protein significantly reduced tumour growth when co‐administered with human PBMCs, highlighting its potential in cancer immunotherapy.^91^ Another TriKE, EGFR/PD‐L1/CD16a, employs a 2+1 common light chain format derived from chicken antibodies, offering a broad epitope spectrum. GTB‐4550, related to PD‐L1, is transitioning from pre‐clinical to clinical trials.^92^

A novel nanoparticle‐based tri‐specific NKCE (nano‐TriNKCE) targeting EGFR‐overexpressing tumours has been developed using a PEG–PLGA matrix, modified with cetuximab and co‐functionalised with anti‐CD16 and anti‐4‐1BB antibodies. Encapsulating the chemotherapeutic drug EPI, these nano‐engagers demonstrated superior efficacy compared with conventional antibodies in vitro by facilitating chemotherapeutic delivery. When combined with NK cell activators and epirubicin, they offer enhanced chemoimmunotherapy potential.^93^ The use of 4‐1BB in this nano‐TriNKCE illustrates how targets from the longer and more extensively studied T cell engagers (TCEs) can be applied to NK cell therapies. This overlap suggests potential for accelerated NKCEs development by leveraging established TCE targets, potentially leading to more comprehensive anti‐tumour strategies.

### 4.5 Clinical implementation of tri‐specific NKCEs

#### 4.5.1 GTB‐3550

The GTB‐3550 TriKE (CD16/IL‐15/CD33), showing promising anti‐tumour efficacy in early studies, led to the launch of a phase I/II clinical trial for treating refractory AML and high‐risk MDS. Recently released clinical data claimed that a surge in NK cell activation was discernible as early as 3 days post‐initiation of the GTB3550 regimen, culminating in a zenith of proliferation by Day 8. Upon completion of the dosing cycle, an expansive upsurge of NK cells was consistently observed across all evaluated subjects.^92^

Additionally, a retrospective glance at the data for continuous infusion of rhIL‐15 divulged a maximum tolerated dose of 2 µg/kg/day, which was previously associated with a suite of side effects.^94^ Nevertheless, strategic alterations in the orientation of IL‐15 within the GTB‐3550 TriKE molecular framework have ameliorated these concerns, as evidenced by the absence of adverse immune responses in patients dosed at 5 and 10 µg/kg/day. Although the initial low‐dose cohorts did not yield discernible objective responses, Subsequent investigations using higher‐dose cohorts will be instrumental in elucidating the therapeutic potential of GTB‐3550 for AML and MDS.^92^

#### 4.5.2 SAR443579

SAR443579 is a novel ANKET (CD123/NKP46/CD16) aimed at treating individuals diagnosed with relapsed or refractory acute myeloid leukaemia. In the Phase 1 trial of SAR443579, the highest non‐toxic dose was 3000 µg/kg, with no dose‐limiting toxicities below this level. However, 95.7% of patients had treatment‐emergent adverse events, 78.3% experienced serious events and 69.6% had treatment‐related events, indicating a notable side effect profile that requires further monitoring. The response rate of 13.0% in a study of patients with relapsed/refractory AML is noteworthy given the challenging nature of treating this condition. Particularly significant is the 37.5% response rate observed in the highest dose cohort, indicating a potential dose‐response relationship, a positive sign in early‐phase trials. Patients typically required a median duration of 16.1 weeks of treatment before observing benefits, suggesting the necessity of sustained treatment for a few months to achieve therapeutic effects. Overall, the clinical trial data for SAR443579 shows promise, especially given the difficult‐to‐treat patient population. The safety profile is manageable, though careful monitoring of adverse events will be crucial. The increased response rate at higher doses indicates potential efficacy. Further investigation in larger trials will be essential to confirm these findings and to determine the therapeutic window for SAR’579 more precisely.^95^

#### 4.5.3 DF1001

DF1001 is a novel tri‐specific NK engager that targets the HER2 antigen by activating NK receptors, particularly NKG2D and CD16. Preliminary investigations have demonstrated that DF1001 induces both direct and indirect robust activation of NK cells, γδ T cells and CD8+ T cells. In the Phase 1 segment of the DF1001‐001 trial, 106 patients with advanced or refractory solid tumours were treated with DF1001 doses ranging from 0.000052 to 15 mg/kg. The treatment was well‐tolerated with no dose‐limiting toxicities, allowing progression to further phases where DF1001 is combined with drugs like nivolumab and nab‐paclitaxel. Pharmacodynamic assessments showed that DF1001 was effective in about 67% of patients, including those with up to 16 prior therapies across various levels of HER2 expression. Impressively, DF1001 registered therapeutic responses in a variety of advanced malignancies, including metastatic breast cancer, colorectal cancer, NSCLC and gastroesophageal cancer. Notably, a response rate of 25% was documented among MBC patients—specifically those with low HER2 expression—even though they had, on average, been subjected to approximately six preceding therapies. DF1001 shows promise as a treatment for advanced malignancies, demonstrating a desirable safety profile and substantial effectiveness in reducing tumour burden in heavily pre‐treated patients. Recently, DF1001 entered phase II clinical trials successfully.^96^

## 5 PERSPECTIVES AND CONCLUSIONS

As cancer immunotherapy evolves, NKCEs are increasingly compared with TCEs, a more established approach. The longer research history of TCE has provided valuable insights for NKCE development. Both have advanced to clinical trials, including BCMA‐targeted therapies (NCT04434469/NCT04557098), each offering distinct advantages. While TCEs have demonstrated high efficacy with approved therapies, NKCEs show promising safety profiles, presenting lower risks of severe cytokine release syndrome and neurotoxicity. NKCEs may offer a safer option for patients with impaired T cell function,^29^, ^67^ despite TCEs currently achieving higher overall response rates in some indications. NKCEs may address some limitations of T cell‐based therapies and be valuable for patients unsuitable for TCEs or in combination strategies to enhance overall treatment efficacy. Despite these comparative advantages, NKCEs face their own unique challenges and opportunities for optimisation. As research in this field progresses, several key areas have emerged as critical for the future development and clinical success of NKCEs.

Previous study showed that TriKE‐associated IFN‐γ levels were observed to surpass those of BiKEs. The elevation in IFN‐γ levels suggests a potentially heightened NK cell activation and more robust anti‐tumour response. Notably, the cytokine release that stimulated by TriKE is still within the physiological ranges, which is essential for therapeutic applications. Over elevated cytokine releases could lead to cytokine storm, and by maintaining levels within physiological limits, TriKEs may offer a safer therapeutic profile. It is important to recognise that while high IFN‐γ levels can indicate a strong immune response, they do not necessarily guarantee therapeutic efficacy. The relationship between IFN‐γ levels and clinical outcomes is complex and may be influenced by various factors. Therefore, although TriKEs show promise in eliciting a strong immune response, further research is needed to fully elucidate their therapeutic potential and optimise their clinical efficacy.

Innate Pharma's tetra‐specific NKCE (IPH6501) features a novel IL‐2 variant, presenting an advanced method for utilising IL‐2 in NK cell therapy.^79^ This dual targeting design not only alleviates the toxicity of IL‐2 but also potentially reduces the impact of IL‐2 stimulation on CD16a expression, addressing several significant limitations of conventional IL‐2 therapy in NK cell activation. Compared with IL‐15‐based TriKEs, these IL‐2 variant molecules may offer better T cell stimulation and more effective modulation of the TME, leading to a more robust and multifaceted anti‐tumour response, which is highly beneficial for cancer immunotherapy.

The suppressive mechanisms within the TME are gaining more attention in cancer research, as they can impede the effectiveness of NK cell‐based therapies. Incorporating TME regulators may be a potential strategy to boost the effectiveness of NKCEs. For instance, combining NKCE therapy with other modalities that modulate the TME or generate a novel subset of NKCEs that target NK cells, tumour antigens and TME modulates such as IL‐33 or TGF‐B to optimise therapeutic outcomes. This can be a reasonable and challenging direction. Presently, the NK receptors involved in NKCEs are predominantly activation receptors; this may stem from the objective of NKCEs, which aims to provoke the proliferation and anti‐tumour activity of NK, targeting the activation receptors in the most direct and efficient way. However, with the forward investigation of NK cells, integrating NK inhibitory receptors into NKCEs may be another potential formulating strategy. NKG2A/HLA‐E axis is known as the immune inhibitory checkpoint for NK cells. Plenty of studies have shown that to escape from the NK cells, some tumour cells overexpress the ligand of NKG2A.^97^ Inhibition of NKG2A has been shown to enhance NK cell‐mediated immunosurveillance.^98^ Among the drugs that targeting NKG2A, monalizumab has emerged as a leading candidate in late‐stage development. Available clinical data and several ongoing clinical trials (NCT05903092, NCT05221840, NCT04307329) indicate that monalizumab holds significant promise, especially when used as part of combination therapeutic strategies.

Another crucial aspect in the development and clinical application of multi‐specific NKCEs is the optimisation of pharmacokinetic properties and delivery strategies. The molecular structure of NKCEs plays a fundamental role in determining their pharmacokinetic behaviour. Smaller formats (e.g., VHH‐based constructs) may offer superior tissue penetration but are susceptible to rapid renal clearance. In contrast, larger structures (e.g., TandAb‐based AFM13 or ANKET‐based SAR443579) tend to have extended circulatory times but may struggle with tumour penetration. Striking a balance between molecular size and functional efficacy remains a significant challenge in NKCE design, necessitating careful optimisation to achieve optimal therapeutic outcomes.

Innovative approaches are being developed to address size‐related limitations in NKCEs. For example, incorporating human serum albumin (HSA) into pure VHH multi‐specific antibodies can extend their half‐life while maintaining the benefits of a compact size.^99^ This highlights the potential for engineered modifications to address size‐related constraints.

The route of administration also impacts NKCE pharmacokinetics. Intravenous (IV) administration provides rapid onset and complete bioavailability but may result in faster clearance. Conversely, subcutaneous (SC) administration provides more sustained plasma levels over time, through with slower absorption.^100^ The choice between IV and SC routes profoundly affects the pharmacokinetic profiles of different NKCEs designs. Larger NKCEs may benefit from IV administration, capitalising on longer circulation times, while SC administration of smaller constructs could extend the effective duration through gradual absorption.

The interplay between molecular structure, target selection and administration route presents both challenges and opportunities for NKCEs optimisation. Emerging approach, such as nanoparticle‐based systems (e.g., nano‐TriKE targeting EGFR/4‐1BB/CD16) may offer exciting possibilities for modulating NKCE pharmacokinetics. These innovations may help reconcile the differences between various administration methods and enable optimised dosing strategies that balance effectiveness, patient comfort and safety.

In conclusion, the future of NKCE therapy lies in the sophisticated integration of molecular engineering, targeted design and innovative delivery strategies. By optimising these factors, more effective, patient‐friendly and versatile NKCEs will be developed, and pave the way for personalised NKCE therapies tailored to specific cancer types and individual patient needs.

## AUTHOR CONTRIBUTIONS

An Zhu wrote the paper. Yu Bai and Yanyang Nan collected data and contributed to the literature search. Yu Bai drew the illustrations. Dianwen Ju edited the paper.

## CONFLICT OF INTEREST STATEMENT

The authors declare no conflict of interest for this paper.

## ETHICS STATEMENT

This review summarises existing literature on NKCEs and their applications in cancer immunotherapy. All referenced studies involving human participants or animals have been conducted in accordance with relevant ethical guidelines and have received appropriate institutional approvals.
